# Did the First COVID-19 National Lockdown Lead to an Increase in Domestic Abuse in the U.K.’s Capital City of London?

**DOI:** 10.1177/08862605241259009

**Published:** 2024-07-24

**Authors:** Chelsea Gray, Kirstine Hansen

**Affiliations:** 1 University College London, UK

**Keywords:** COVID-19, national lockdown, domestic abuse, victimization

## Abstract

On March 23, 2020, the United Kingdom went into national lockdown to stop the spread of COVID-19. In this paper, we examine whether a policy aimed at minimizing the health consequences of the pandemic had unintended negative consequences for domestic abuse. Using data from the Metropolitan Police in England we estimate the impact of lockdown on domestic abuse in the 32 boroughs that make up the London metropolitan area. Using a before and after approach, and controlling for other factors, we show an increase in the probability of being a victim of domestic abuse during lockdown similar in magnitude to the increase experienced over the Christmas holidays. However, the overall picture masks inequalities across groups: with women, younger and older people, and people of Asian, Arab, and Middle Eastern ethnicity subject to the highest increases, reflecting vulnerabilities and existing inequalities. Of the domestic abuse-related crimes, it is the most violent crimes that saw the greatest increases during lockdown. Once lockdown restrictions are eased, rates decline but remain slightly higher than prior to lockdown up to 3 months later. The results present a clear message for policy makers: a policy adopted to alleviate one problem, even in times of crisis, must factor in the impact this may have in other areas. Failure to do so in this situation, despite existing evidence linking domestic abuse to stress, confinement, and crisis situations prior to lockdown, has resulted in an increase in domestic violence in the U.K.’s capital city, during lockdown and beyond.

When the prime minister of the United Kingdom, Boris Johnson, addressed the nation on March 23, 2020, to tell viewers the country was going into lockdown, it became one of the top 10 most-watched broadcasts in history, attracting 27.5 million viewers (Duncan, 2020). The restrictions put in place meant that people could only leave their homes to shop for necessities, as infrequently as possible, exercise for up to an hour a day, and only travel to work if their job could not be conducted from home. Non-essential shops were forced to close, any social events or gatherings (such as weddings, christenings, or birthdays) were banned, schools were shut, and sports events were canceled. These restrictions were set in place in the United Kingdom, along with other countries,^1^ to stop the spread of COVID-19. These restrictions stayed in place for seven full weeks of lockdown, until conditions were eased on May 10th when people were allowed to return to work and take unlimited exercise, and finally, lockdown restrictions were fully lifted on June 15th when non-essential shops and primary schools were re-opened.

While this measure was designed to save lives and alleviate pressure on the National Health Service (NHS) there was little or no discussion at this stage of the effect this confinement would have on domestic abuse, despite the fact that existent evidence showed domestic abuse tends to increase when families spend more time together (like Christmas) (Taub, 2020) and in stressful situations (Flynn & Graham, 2020). In the 12 months ending March 2019, there were an estimated 2.4 million adults in the United Kingdom, who were victims of domestic abuse (Office of National Statistics, 2019). For these people, home is far from a safe place and often the freedom of leaving home to go to work or visit family and friends is an escape from their abuser (Mitchell & Hodson, 1983). This paper uses data from the Metropolitan Police for the whole of the metropolitan area of London to examine what happened to domestic abuse before and after the first COVID-19 national lockdown and beyond. Specifically, we examine three hypotheses: (a) Did domestic abuse increase during lockdown, (b) were changes homogenous across different population groups and different crimes, and (c) were the changes homogenous during lockdown and did they revert to pre-lockdown levels after lockdown eased?

## Background

Domestic abuse can be committed by both men and women and is defined as “an incident or pattern of incidents of controlling, coercive, threatening, degrading and violent behaviour” (Davidge et al., 2020).^2^ It is used as an umbrella term, capturing all inter-partner violence and domestic violence within the family. It is estimated that around 40,000 calls were made to Domestic Violence charities in the initial 3 months of lockdown, which is almost an 80% increase from the norm (Kelly & Graham, 2020). Newspapers were reporting that other countries were also seeing increases in domestic abuse including Brazil, Belgium, and Italy (Graham-Harrison et al., 2020), and that this was a global occurrence (Gulland et al., 2020).

Such concerns led a number of people to speak out about a potential link between lockdown and an increase in domestic abuse prior to the United Kingdom going into lockdown. Indeed, in a statement made by Dr Hans Kluge the director of the World Health Organization European region on March 4, 2020, 19 days before the United Kingdom went into lockdown, he talked about increases in domestic abuse and violence against women occurring across Europe “as women are forced to ‘lockdown’ at home with their abusers.” At the same time, he acknowledged that access to services in some European areas was being disrupted due to COVID-19, with shelters either full, repurposed for COVID-19 reasons or closed (World Health Organization, 2021).

With evidence from countries that entered lockdown before the United Kingdom, the New York Times reported they contacted the U.K. Home Office 10 days prior to lockdown to ask what the United Kingdom was going to do about domestic violence. Domestic abuse charities sent an open letter to Parliament stating that emergency measures would need to be put in place to combat the rise in domestic abuse (Home Office, 2020). And on April 5, 2020, António Guterres, the Secretary General of The United Nations, wrote a Tweet calling for “governments to put women’s safety first as they respond to the pandemic.”

### Conceptualizing the Link Between COVID-19 and Domestic Abuse

To help us think about why lockdown may lead to an increase in domestic abuse we start with the general criminological theories that suggest situation and interaction are crucial for crime (Campedelli et al., 2021). Key here are Routine Activity (Cohen & Felson, 1979) and Crime Pattern Theories (Brantingham & Brantingham, 1984) which predict crime will occur where a motivated offender’s space intersects the space of a suitable target, and in the absence of a capable guardian, this results in a crime. These theories would predict an increase in domestic abuse during lockdown when people are confined to the home, as long as there is a motivated offender. To help us think about how lockdown would increase motivation we draw specifically on theories relating to domestic abuse, in particular, the work of Flynn and Graham (2010) who, from their systematic review of the domestic abuse literature, categorize the reasons for domestic abuse into three levels: Level 1 relates to background and personal attributes; level 2 to current life circumstances; and level 3 to immediate precursors or precipitators. Level 2 is fundamental to this research because all the drivers of what Flynn and Graham (2010) refer to as life circumstances, including health, finances, physical and mental well-being, and marital and family worries are likely to be negatively affected by COVID-19 and lockdown.

With some cross-over with Flynn and Graham’s level 2 factors, Peterman et al. (2020) offer a pathway model that directly addresses how pandemics might affect violence against women and children which includes factors such as economic insecurity and poverty-related stress; isolation and quarantine; disaster-related unrest and instability; exposure to exploitative relationships due to changing demographics; reduced health service availably; and inability of women to escape abusive partners which act in direct and indirect ways and interact, reinforcing existing inequalities.

Informed by these theories and typologies we can see how COVID-19, which quickly changed the economic environment for families, with increasing financial insecurity and financial-related stress, factors which are known predictors of domestic abuse (Benson et al., 2003), could lead to an increase in domestic abuse. During the spring and summer months of 2020 estimates from the Office of National Statistics show that around 7.5 million people, more than a quarter of the workforce, were temporarily away from work during the lockdown period (Office of National Statistics, 2020): By July 2020 Universal Credit claims^3^ had risen by around 117% to 2.7 million; regular nominal pay had fallen for the first time since records began in 2001; and business’ had secured government-backed loans of almost £52 billion with 9.6 million workers’ pay supported through the Job Retention Scheme (Sky News, 2020).

Lockdown restrictions, which confine people to the same household space, could exacerbate existing conflicts or create conflict where none existed: increasing family stress, which even without lockdown has been cited as a key motivator behind domestic abuse (Cascardi & Vivian, 1995; Conger et al., 2000). Previous health emergencies, including the 2003 Severe Accute Respiratory Syndrome (SARS) outbreak have been associated with increased anxiety, and mental and physical health problems including post-traumatic stress, depression, and even suicide attempts (Peterman et al., 2020), with quarantines and social isolation identified as possible contributing factors (Lau et al., 2005; Reissman et al., 2006; Yeung & Fung, 2007).

Peterman et al.’s (2020) typology also encourages us to draw on evidence from work that examines the aftermath of disasters (including hurricanes, tsunamis, floods, wildfires, and volcano eruptions), when thinking about how COVID-19 may impact domestic abuse. This literature fairly consistently shows that any major event that puts a restriction on people and places is associated with an increase in domestic abuse and violence (Anastario et al., 2009; Chew et al., 2005; Dobson,1994; Fothergill, 1998; Klein, 2008). In particular, the findings show that those most affected tend to be women, children, and other vulnerable people (Phillips et al., 2009) who are disproportionally victims of domestic abuse in general, and who experience the highest increases in times of crisis (Dasgupta et al., 2010; Phillips et al., 2009).

### Evidencing the Link Between COVID-19 and Domestic Abuse

Early empirical evidence on the impact of COVID-19 social restrictions and lockdown policies on domestic abuse primarily addresses issues relating to the overall impact that lockdown had on domestic abuse. With a growing number of studies using data on domestic abuse calls to the police, crimes, calls to service or domestic abuse helplines, and hospital reports,^4^ painting a complex pattern from different locations around the world.

On the whole, there is more evidence in support of lockdown leading to an increase in domestic abuse than not. Of the studies that examine more than one source most find more evidence of domestic abuse increasing with lockdown. Brink et al. (2021) and McNeil et al. (2023) in cross-country comparisons found 60% of countries examined showed a positive relationship between lockdown and domestic abuse. In studies of multiple large U.S. cities Boserup et al. (2020), Leslie & Wilson (2020), and McCrary and Sanga (2021) showed increases in domestic abuse over lockdown. While, Kofman et al. (2023) found mixed results in Orange County, with increases in Santa Ana but no change in Anaheim.^5^

Of the single focus studies those examining calls to helplines show a strong consensus that the number of calls about domestic abuse increased during lockdowns around the world (see Agüero (2021) for evidence from Peru; Gibbons et al. (2020) and Perez-Vincent and Carreras (2020) for Argentina; Home Office (2020) for the United Kingdom; Silverio-Murillo and Balmori de la Miyar (2020) for Mexico City, Mexico; Weller et al. (2021)for London and Surrey, UK; and Women’s Safety NSW and Foundation for Alcohol Research & Education (2020) for New South Wales, Australia). Studies using reports of domestic abuse to the police produce more mixed results: studies in Los Angeles and Indianapolis (Mohler et al., 2020), Chicago (Bullinger et al., 2021), New Orleans (Shariati & Guerette, 2022), Dallas (Piquero et al., 2020), and India (Ravindran & Shah, 2020) all found lockdown increased reports/calls to the police. Although, Capinha et al. (2021) found a decrease in calls for service in Portugal and studies using reported crimes in Los Angeles (Campedelli et al., 2021), Lancashire, UK (Halford et al., 2020), and New South Wales, Australia (Freeman & Leung, 2020) likewise found no increase in domestic abuse.

There is less evidence of differences across groups, although in the United Kingdom Ivandić et al. (2020), present evidence from the London metropolitan area, using calls for service matched data to the relationship between victim and abuser to show that during the COVID-19 lockdown patterns of abuse varied significantly by the type of perpetrator: With lockdown leading to a rise in abuse by current partners and family members of 8% and 17% but a decrease of 11% in abuse by ex-partners.

Evidence on the timing or longevity of results is also currently sparse. Although Shariati and Guerette (2022) showed that while domestic abuse calls increased in New Orleans, they did not do so universally across the lockdown period; Piquero et al. (2020) found a spike in domestic abuse after the lockdown in Dallas but showed that the increase was short-lived; and Kofman et al. (2023) found increases in parts of Orange County that lasted up to 54 weeks after lockdown.

What is emerging is a picture from around the world of the complex relationship between domestic abuse and lockdown. What is clear is that because most of the evidence comes from a single hospital, service provider, police force, or geographical location we are not comparing like with like across these studies as other things vary across these areas that will affect results such as area characteristics (Kofman et al., 2023), intensity of lockdown, the timing and length of lockdown, and policies and support available for domestic abuse victims (Brink et al., 2021). Such differences also mean there are concerns around the generalisability, or external validity, of individual results. For this reason, it is crucial that we build a complete picture of the situation across as many different areas as possible, so we are able to compare different results across different areas to better understand potential mechanisms that lie behind the relationship between COVID-19 and domestic abuse.

To this end, we set out to address our three hypotheses using data from the largest metropolitan area in the United Kingdom: (a) Did domestic abuse increase in London during lockdown?; (b) Were changes homogenous across different population groups, and different crimes?; and (c) Were the changes homogenous during the whole lockdown period and did they revert to pre-lockdown levels after lockdown eased? Our first hypothesis adds to the existing literature on how lockdowns affected the lives of people around the globe. In this paper we are able to say something about the size of the problem in London, showing how the probability of being a victim of domestic abuse varies in lockdown compared to previous periods/years. With our second hypothesis, we are also able to say something about how the relationship between lockdown and domestic abuse varies across different types of populations, showing the groups most at risk of domestic abuse during lockdown and how these map onto existing inequalities. And finally, in addressing our third hypothesis we can talk about whether domestic abuse varies across the duration of lockdown and what happens after lockdown has been eased.

## The Current Study

To answer these questions, we use a before and after methodological approach to examine the probability of being a victim of domestic abuse in the months before and after lockdown. Controlling for other variables, and setting up a placebo test using the same time frame but in the year before lockdown, when no lockdown was in place, our methodology allows us to better attribute any changes in domestic abuse to lockdown. We then go on to examine the crimes and populations that saw the highest increases in domestic abuse over the lockdown period before turning our attention to what happens after lockdown using an event study approach.

## The Data

**Figure 1.**
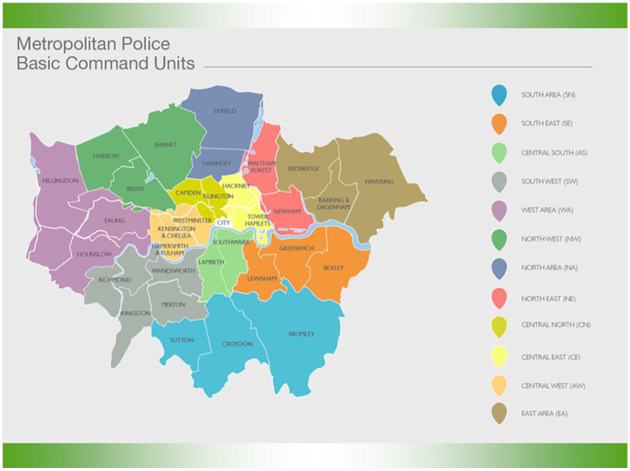
The London metropolitan area, dividing into 12 Basic Command Units.

The data used in this analysis come from the Crime Reporting Information System (CRIS) used by the Metropolitan Police Service to record all crimes within the 32 London Boroughs, which make up 12 Basic Command Units (BCUs) (Figure 1).

The Metropolitan Police define domestic abuse as:

Any incident of threatening behaviour, violence or abuse (psychological, physical, sexual, financial or emotional) between adults, aged 16 and over, who are or have been intimate partners or family members, regardless of gender and sexuality.^6^

Incidents can be reported in a number of ways^7^ and are recorded as crimes by the responding officers.

The data used in this analysis are recorded crimes, by the day, by crime type, by London Borough, and for the majority of observations, by the ethnicity, gender, and age of the victim. These population-level data, measured over time, ensure high external validity and give us confidence that our results are generalizable for the area we examine. Within the data we examine there are various types of crimes that are recorded as domestic abuse. Table 1 shows the domestic abuse crimes recorded in the 12 weeks of lockdown and the 11 weeks prior to that,^8^ by type of offense. During this period there were 281,223 offenses reported in all, of those 40,118^9^ or 14.3% were recorded as domestic abuse incidents. The results show that although the numbers across all crime types look higher in the lockdown period, the types of offense, as a percentage of all domestic abuse crimes, are similar across both periods. In both periods the majority of domestic incidents (87%) are violent crimes, with common assault accounting for 26% of all domestic crimes in the 11 weeks prior to lockdown and 28% in the lockdown weeks, closely followed by assault with injury (accounting for 21% and 23% across the two time periods) and harassment (23% and 20% across the two time periods).

**Table 1.** Numbers of Domestic Abuse Crimes by Type in Lockdown Compared to the 11 Weeks Prior to That.

By Offense	11 Weeks Prior to Lockdown	Lockdown Period
Violent		
Common assault	4,468 (26.28)	6,410 (27.71)
Assault with injury	3,518 (20.70)	5,287 (22.85)
Harassment	3,855 (22.70)	4,612 (19.94)
Serious wounding	1,282 (7.55)	1,761 (7.61)
Other violence	900 (5.30)	1,238 (5.35)
Rape and other sexual offenses	727 (4.28)	759 (3.28)
Arson	15 (0.09)	44 (0.19)
Murder	4 (0.02)	5 (0.02)
Non-violent		
Criminal damage	1,028 (6.05)	1,597 (6.90)
Theft	847 (4.99)	1,052 (4.55)
Burglary	178 (1.05)	189 (0.82)
Other	168 (0.99)	179 (0.77)
Total	16,985 (100.00%)	23,133 (100.00%)

*Note.*Table displays numbers, then shows as a percent of all domestic abuse crimes in parentheses.

## Methods

**Figure 2.**
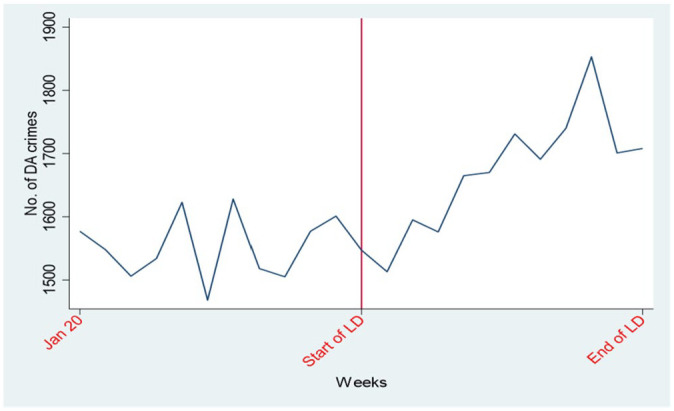
The number of domestic abuse crimes prior to, and during, lockdown.

Our first research hypothesis is that domestic abuse is likely to have risen during the lockdown period. If we plot the number of domestic abuse crimes from January 2020 through the lockdown period (Figure 2) we can see that the raw numbers seem to suggest an increase in domestic abuse over lockdown.

To test this more formally, we employ a before and after approach. In this framework, the probability of being a victim of domestic abuse in the weeks during the COVID-19 lockdown is compared to the 11 weeks prior to lockdown. If the research hypothesis is correct, we expect to see an increase in domestic abuse after March 23rd.

The model takes the simple form of:

$$Pr{\left(Y=1\right)}_{\mathrm{it}}=\beta_{t}(\mathrm{lockdown})+\varepsilon_{\mathrm{it}}$$

where *Y* is the probability of being a victim of domestic abuse, β_t_ is a dummy indicating the lockdown period, compared to a base which is the period prior to lockdown. This coefficient essentially encapsulates the effect of lockdown on the probability of being a victim of domestic abuse, or the causal impact of COVID-19 lockdown on domestic abuse if there is nothing else going on at the same time that would impact on the incidence of domestic abuse, a matter we discuss in more detail below. What we are looking for is whether the coefficient is positive and significant in the lockdown period and the size of the effect; indicating that domestic abuse rose after lockdown (and by what amount).

The analysis controls for a range of factors that may account for any differences in domestic abuse. The CRIS data, in most cases, has the ethnicity, gender, and age of victim, which are added to the models to control for victim characteristics. Additionally, area-level controls are added in the form of dummies for the 12 Basic Command Unit areas.^10^ These are essentially, area fixed effects, controlling for unobservable factors that may impact domestic abuse within an area but do not vary, or vary very minimally, across time. Finally, we control for crime type within the domestic abuse crimes (as we have shown some crimes are more associated with domestic abuse than others) and changes in crime across the period as we are aware that COVID-19 and lockdown saw a shift in the patterns of crime we are used to seeing (BBC News, 2020; Kirchmaier & Villa Lera, 2020).

While the external validity of our approach is high, like all before and after analyses, where there is no control group, the internal validity of our results, or our ability to interpret our results in a causal way, may be weakened by things that are happening at the same time as lockdown which offer possible alternative explanations for our observed results. In this case, despite the fact that we are looking over a relatively short period of time, there are a few possible sources of threat. The first is that domestic abuse may be seasonal so any increases we see over the lockdown period, may occur every year over the same period. To deal with this threat we set up a placebo test, examining the same time frame the year before, when no lockdown was in place. This ensures that any differences that are found over the lockdown period are not the result of seasonal trends in domestic abuse but are, in fact, a result of the national COVID-19 lockdown.

A second potential threat to internal validity comes from the possibility that during lockdown police resources or focus shifted from other crimes toward domestic abuse. In this case, any rise in domestic abuse crimes would be attributable to an increase in police targeting domestic abuse rather than a genuine increase in domestic abuse. But the Metropolitan Police did not have a shift in policy toward domestic abuse over the lockdown period. So, this is unlikely to challenge the internal validity of our analysis. However, lockdown did see a decline in a number of types of crime including theft and burglary, which have a high offense count and are little associated with domestic abuse (see Table 1), which, according to our data produced a reduction of around 4,500 crimes of these types by June 2020, compared to March 2020. This is unlikely to have been totally offset by the increase in the types of crimes that rose over lockdown such as drug offenses and public order offenses (which combined produced an increase of around 2,000 crimes of this type by June 2020 compared to March 2020 in London). So, we cannot fully rule out police being better able to respond to domestic incidents due to less demand on their time in other areas.

Finally, a third and related threat comes from the fact that during lockdown more people were at home which could lead to an increase in calls to the police about domestic abuse incidents from neighbors. Ivandić et al. (2020) show that lockdown saw an increase in calls about domestic abuse in London. Most of the increase was an increase in calls by third parties. In this analysis we are looking only at domestic abuse crimes, and while calls about domestic incidents may increase with more people at home this does not necessarily lead to more recorded domestic abuse crimes. The data we are using show that from January 2020 to August 2020, the Metropolitan Police received just over 50,000 calls from third parties about domestic abuse and just under 50,000 calls from victims of domestic abuse. Of the calls made by a third party only 17% end up recorded as domestic crimes, compared to 30% of calls made by victims. The data we use in this analysis are recorded crimes, not reported crimes.

Having done all we can to reduce the threats to the internal validity of our approach, after testing whether lockdown is associated with an increase in domestic abuse in general, our second hypothesis is tested by adding interactions to our models to allow the lockdown period to vary by sub-groups of the population. This tells us whether the impact of lockdown was felt more heavily among certain groups. We focus on sex, age, and ethnic group differences as well as differences in offense type. Finally, using an event-type approach, we move from a single before and after period to an examination of trends by week to assess whether the impact of lockdown varies over the duration of lockdown and look at what happens after lockdown restrictions are eased.

## Results

To examine whether the impact of the national lockdown led to an increase in domestic abuse, Table 2 shows the average marginal effects (AME) of a probit model^11^ examining the probability of being a victim of domestic abuse during the 12 weeks of lockdown, compared to the 11 weeks before. The coefficient for the lockdown period shows that there is a positive significant relationship, indicating that individuals are 6.6 percentage points more likely to be a victim of domestic violence during lockdown compared to the weeks before, with the probability of being a victim of domestic abuse increasing from 11% to 17.7% during lockdown.

**Table 2.** Average Marginal Effects on the Probability of Being a Victim of Domestic Abuse Before and After the COVID-19 Lockdown.

After (March 23 to June 15, 2020) Before (January 7, 2020 to March 23, 2020)	AME (d*y*/d*x*)
Lockdown	0.066*** (0.001)
*N*	264,263
Pseudo *R*^2^	0.0115

*Note.* Coefficients is a dummy variable for the lockdown period compared to the period prior to lockdown showing the average marginal effects from a probit model. Robust standard errors are in parentheses.

* *p* < .05. **p < .01. ***p < .001.

### Adding Demographic Controls With Crime and Area Fixed Effects

The results show the probability of being a victim of domestic abuse increased over the lockdown period compared to the period prior to lockdown. However, so far, we have not included in our models any controls for other things that might affect the relationship of interest. Therefore, like Table 2, Table 3 uses the 11 weeks before lockdown as the base and displays the results for the 12 weeks of the first national lockdown. Though this time, the model controls for a range of other factors: Model A controls for the demographics of the victim and accounts for the sex of the victim, their age, and ethnicity as previous research has shown that domestic abuse victimization is likely to occur differentially across these groups (Gill et al., 2012). Model B controls for the area in which the crimes occurred. Adding area dummies for the 12 Basic Command Units (BCUs) allows us to take account of factors that are different across areas but that remain constant, or almost constant (as we are looking at such a short period) across time, for example, the fact that some areas of London always have higher crime rates than others or have different population densities. In model C, dummies are added to account for crime type as we showed in Table 1 certain crimes are more associated with domestic abuse than others. All the controls are displayed together in model D, which also includes interactions between all the control variables and the after period. This allows the association between the control variables and our outcome to vary before and after lockdown.

**Table 3.** Average Marginal Effects of Being a Victim of Domestic Abuse During Lockdown, Controlling for Victim Demographics and Area and Crime Fixed Effects.

	AME (d*y*/d*x*)			
	Demographics	Area Controls	Crime Type	All Controls (Incl. Interactions)
Variables	A	B	C	D
Lockdown	0.056*** (0.001)	0.061*** (0.001)	0.030*** (0.001)	0.025*** (0.001)
Demographics	Yes	No	No	Yes
Area controls	No	Yes	No	Yes
Crime controls	No	No	Yes	Yes
All two-way interactions between controls and lockdown period	No	No	No	Yes
*N*	264,263	264,263	264,263	264,263
Pseudo *R*^2^	0.149	0.021	0.206	0.294

*Note.* The coefficients are dummy variables for the lockdown period compared to the 11 weeks prior to lockdown (to exclude the Christmas period). They are average marginal effects from a probit model. The controls are gender of victim, ethnicity, age of victim, 12 BCUs, crime type, and interactions between each control and the after period. Robust standard errors in parentheses.

* *p* < .05. **p < .01. ***p < .001.

Without the controls, the previous results show almost a 7 percentage point increase in the probability of being a victim of domestic abuse during lockdown. Including the controls reduces the coefficients by varying measures. Including area controls (model B) has the least effect on the magnitude of the coefficient of interest, which remains at 6.1 percentage points and only accounts for 2% of the variation in our outcome. Including the demographic information on victims (model A) decreases the initial 7 percentage point increase we were seeing in the probability of being a victim of domestic abuse over lockdown by slightly more, with the increase now at 5.6 percentage points. This model accounts for around 15% of the variation in probability of being a victim of domestic abuse. Including crime type cuts the post-lockdown increase further to 3 percentage points and accounts for more variation in the outcome (21%). The final model, which includes all control variables along with interactions between them and the after period again slightly reduces this coefficient to 2.5 percentage points and accounts for just under 30% of the variation in the outcome.

### Placebo Test: The Same Analysis the Year Before

The results so far suggest that even after controlling for victim demographics, area, and crime-fixed effects and allowing all control variables to interact with the lockdown period there is a small positive impact of the COVID-19 first national lockdown on domestic abuse. However, we are also aware that crime is seasonal. So there remains a possibility that the positive results shown during the lockdown period are actually reflecting a seasonal trend, rather than a genuine increase in domestic abuse resulting from lockdown. To test this, we run a placebo test, the same model as Table 3, with full controls, this time though for the exact same time period the previous year, when COVID-19 did not exist and the country was not in a national lockdown. The results, displayed in Table 4, show a very slight decline in the probability of being a victim of domestic abuse over the period examined, although this is only statistically significant in one of the models. This gives us confidence that the increase in domestic abuse during the first national lockdown in 2020 is a result of the COVID-19 lockdown rather than a seasonal trend.

**Table 4.** Average Marginal Effects of Being a Victim of Domestic Abuse in the Year Prior to COVID-19, Controlling for Victim Demographics and Area and Crime Fixed Effects.

	AME (d*y*/d*x*)			
	Victim Demo	Area Controls	Crime Type	All Controls (Incl. Interactions)
Variables	A	B	C	D
Placebo lockdown	0.001 (0.001)	−0.002 (0.001)	−0.003*** (0.001)	−0.002 (0.001)
Victim demographics	Yes	No	No	Yes
Area controls	No	Yes	No	Yes
Crime controls	No	No	Yes	Yes
All two-way interactions between controls and lockdown period	No	No	No	Yes
*N*	323,322	323,322	323,322	323,322
Pseudo *R*^2^	0.134	0.018	0.22	0.309

*Note.* The coefficients are dummy variables for the lockdown period compared to the 11 weeks prior to lockdown (to exclude the Christmas period) but in the year before lockdown. Controls—as for Table 3. Robust standard errors in parentheses.

* *p* < .05. **p < .01. ***p < .001.

### Do All Types of Domestic Abuse Crimes Increase During Lockdown?

We have already seen the importance of controlling for crime type and the changing patterns of crime over lockdown. For obvious reasons lockdown saw a decline in crimes such as theft and burglary while others such as drug-related crimes and public disorder offenses increased during lockdown (BBC News, 2020; Kirchmaier & Villa Lera, 2020). In this section we examine the probability of being a victim of specific types of domestic abuse within the key crime types that were identified in Table 1^12^ as those most associated with domestic abuse both before and after lockdown. To do this we display coefficients that are average marginal effects from an interaction term between each type of domestic abuse crime and the lockdown period from the same model that was used for the final specification in Table 3. The coefficients are thus interpreted as the movement in the predicted probability of being a victim of each type of domestic abuse crime and measured in the usual way in percent point terms. What we are interested in is whether all crime types have coefficients of a similar magnitude, indicating that the increase in domestic abuse was similar across all domestic abuse offenses, or whether the coefficients vary, indicating the rise in domestic abuse is associated with some offenses more than others. What we can see from column 1 in Table 5 is that the most violent categories of domestic abuse were the ones that saw the largest increases during lockdown, the probability of being a victim of domestic abuse-related assault with injury increased after lockdown by nearly 8 percentage points, common assault by just over 5 percentage points and serious wounding by 4 percentage points.

**Table 5.** Average Marginal Effects of Being a Victim of Domestic Abuse for Each Type of Crime After Lockdown (With Full Controls).

Offense	AME (d*y*/d*x*)	
	Probability of Being a Victim of Domestic Abuse for Each Type of Crime After Lockdown	Probability of Being a Victim of Each Type of Crime After Lockdown for Everyone^13^
Violent		
Common assault	0.054*** (0.005)	0.028*** (0.001)
Assault with injury	0.078*** (0.006)	0.019*** (0.001)
Harassment	0.001 (0.004)	0.052*** (0.001)
Serious wounding	0.044*** (0.008)	0.007*** (0.001)
Other violence	0.017* (0.009)	0.012*** (0.001)
Rape and other sexual offenses	0.028*** (0.007)	0.001 (0.001)
Non-violent		
Criminal damage	0.039*** (0.005)	0.011*** (0.001)
Theft	0.018*** (0.001)	−0.097*** (0.002)
Burglary	0.007*** (0.002)	−0.017*** (0.001)
Full controls	Yes	Yes
*N*	264,263	264,263
Pseudo *R*^2^	0.293	0.027–0.140

*Note.* The coefficients in column 1 are interaction terms between crime type and the after period and are average marginal effects from a probit model that includes a full set of controls. The coefficients in column 2 are the average marginal effects of being a victim of that type of crime in general in the lockdown period. The models are run separately for each crime type, which is why there is no one Pseudo *R*^2^ value but a range. All models include a full set of controls for gender of victim, ethnicity, age of victim, 12 BCUs, crime type, and interactions between each control and the after period. Robust standard errors in parentheses.

* *p* < .05. **p < .01. ***p < .001.

We can compare these increases to the movement in the probability of being a victim of these same offenses in general after lockdown by comparing column 1 to column 2. When we do this, we can see even in instances where the increase in domestic abuse looks relatively low such as rape and sexual offenses the small post-lockdown rise in domestic abuse-related rapes and sexual offenses contrasts with the lack of increase in these types of offenses in general. For domestic abuse-related theft and burglaries, the very small increases in domestic violence-related offenses contrast to the general downward trend in these offenses in general post-lockdown, which saw thefts decrease by around 10 percentage points and burglary by just under 2 percentage points.

### Which Populations Were Most Affected by the Increase in Domestic Abuse Due to Lockdown?

Having established a link between lockdown and an increase in domestic abuse, and having shown that this increase was not homogenous across all types of domestic abuse offenses, we now turn our attention to examine whether the increase was experienced homogenously across different populations. We already know that certain populations are more likely to be victims of domestic abuse in non-crisis situations and that in crisis situations these groups are the most affected by increases in violence (Dasgupta et al., 2010; Phillips et al., 2009) which leads us to expect that the impact of the lockdown will have been experienced differentially across different populations, with the most vulnerable groups seeing a greater increase than other groups. This is exactly what the analysis finds: Based on the full model with all controls (in Table 3), Table 6 shows the coefficients of the interactions between the demographic characteristics and the lockdown period, showing the predicted probability of being a victim of domestic abuse during lockdown compared to the previous period for each population group. The results show that, as hypothesized, some groups experience a larger increase in the probability of being a victim of domestic abuse after lockdown than others. Women, who already experience more domestic abuse than males, experience a greater increase after lockdown (almost 4 percentage point increase compared to the 1.6 percentage point increase experienced by males). When we look at the different age groups, we see the largest rise is among younger people (16–21) and older people (70+). Both groups see just over a 4 percentage point increase in the probability of being a victim of domestic abuse during lockdown. For other age groups, the corresponding rise is around 2 percentage points. When the ethnicity of the victim is examined, Asians and those of Arab and Middle Eastern ethnicity see the largest increase in the probability of being a victim of domestic abuse during the first national lockdown, with an increase of around 6 percentage points compared to a rise of 3.7 percentage points for Black people and 2.7 percentage points for white people.

**Table 6.** Average Marginal Effects of Being a Victim of Domestic Abuse After Lockdown, by Victim Demographics (With Full Controls).

Demographic Variables	AME (d*y*/d*x*)
Gender	
Female	0.039*** (0.002)
Male	0.016*** (0.001)
Age	
≤21	0.041*** (0.002)
22–29	0.024*** (0.003)
30–39	0.026*** (0.002)
40–49	0.018*** (0.003)
50–59	0.022*** (0.003)
60–69	0.015** (0.006)
70+	0.042*** (0.007)
Ethnicity	
Black	0.037*** (0.004)
Arab/Middle Eastern	0.060*** (0.010)
Asian	0.056*** (0.004)
White	0.027*** (0.002)
Full controls	Yes
*N*	269,648
Pseudo *R*^2^	0.292

*Note.* The coefficients are average marginal effects from a probit model that includes a full set of controls for gender, ethnicity, and age of victim, 12 BCUs, crime type, and interactions between each control and the after period. Robust standard errors in parentheses.

* *p* < .05. **p < .01. ***p < .001.

### Allowing for Heterogeneity in the Lockdown Period and Beyond: An Event Study Approach

So far, the analysis has only considered the impact the first national lockdown had on the probability of being a victim of domestic abuse before and during the lockdown period. In the final part of the analysis, we turn our focus to whether the increase we have seen in domestic abuse in the lockdown period is homogenous across the whole lockdown period or whether domestic abuse increases with the duration of lockdown, before turning our attention to what happens after lockdown restrictions are eased on May 10th (when people were allowed to return to work and take unlimited exercise) and finally when lockdown restrictions are fully lifted on June 15th (when non-essential shops and primary schools were re-opened). We might expect that once lockdown restrictions are eased, and then finally lifted, the incidence of domestic abuse will return to the levels seen prior to lockdown. However, research looking at the impact of terrorist acts on hate crimes shows that hate crimes increase after terrorist acts and do not return to lower levels for long periods afterwards (Ivandić et al., 2019) and research on COVID-19 shows hate crimes against Chinese people increased and remained at high levels 8 months after COVID-19 first emerged (Gray & Hansen, 2020). So, following on from this, we may expect domestic abuse levels to remain higher after lockdown: As while the people have freedom to leave the house, the structural stresses and fears associated with the pandemic may remain.

**Figure 3.**
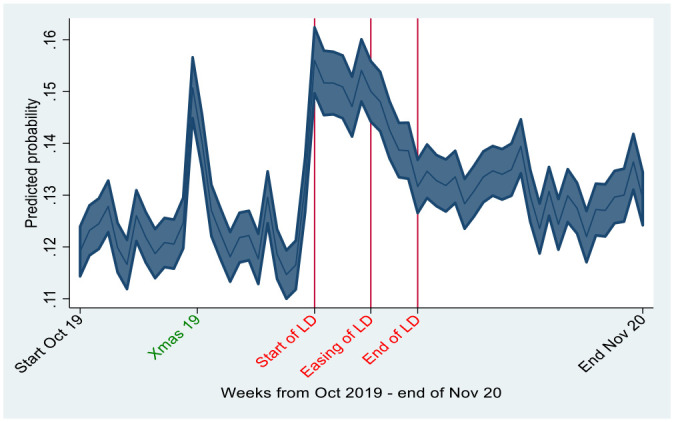
Predicted probability of being a victim of domestic abuse before during and after lockdown.

Both of these aspects are considered in Figure 3 which plots the predicted probability of being a victim of domestic abuse by week from October 2019, prior to COVID-19, into 2020 when COVID-19 emerged, through the first national lockdown, and beyond its removal through to the end of August 2020. The graph shows no significant movement in the predicted probability of being a victim of domestic abuse prior to lockdown; remaining steadily around 12% (except for a rise over the Christmas period to around 15% in line with evidence from Taub (2020)). In the week before lockdown the probability increased to around 14%, which coincides with the week Boris Johnson began his daily COVID-19 press briefings on March 16th urging everybody in the United Kingdom to work from home and avoid pubs and restaurants prior to the formal lockdown command on the evening of March 23rd.^14^ There is a further significant increase as we move into the first week of lockdown to around 16%, with the probability staying between 15% and 16%, similar to the probability of being a victim of domestic abuse over the Christmas period, for the duration of lockdown.

As soon as lockdown is eased the predicted probability of being a victim of domestic abuse declines slightly dropping to 13% by the end of lockdown. However, it remains around 13% to 14%, slightly higher than the probability experienced prior to lockdown which was just under 12%. A similar picture emerges if we look at this by gender, age, and ethnicity (not shown). With the exception of the Christmas period, the predicted probability of being a victim of domestic abuse remains relatively stable until just prior to lockdown when it starts to rise and remains significantly higher throughout lockdown, declining when restrictions are eased, but remaining slightly higher 3 months after lockdown than prior to lockdown.

## Discussion

This paper set out to test whether the first U.K. COVID-19 national lockdown (between March 23, 2020 and June 15, 2020) was associated with an increase in domestic abuse in the London metropolitan area. Using a before and after approach we examined whether domestic abuse was higher during lockdown than it was before and showed that even after controlling for other factors associated with differences in domestic abuse there is a rise in recorded domestic abuse crimes during lockdown, when the probability of being a victim of domestic abuse increased, by around 3 percentage points, on average. In terms of magnitude, the increase is similar to the rise in domestic abuse associated with the Christmas holidays. When we ran a placebo test for the year prior to COVID-19 and lockdown we found no similar significant increase in domestic abuse, giving us confidence that the increase we found was a result of the national lockdown and not some seasonal trend.

The second research question focuses on whether the increase in domestic abuse is greater for some groups than others, with results showing that women, the youngest age group, and older people experienced higher increases in the probability of being victims of domestic abuse during lockdown than others. Increases were also higher for people of Asian, Arab, and Middle Eastern ethnicity, who saw increases of around 6 percentage points in their predicted probability of being a victim of domestic abuse during lockdown. Of the domestic abuse-related crimes, it was the most violent crimes that saw the greatest increase during lockdown.

Finally, when we allowed the lockdown period to have a different impact on domestic abuse across the duration of lockdown, to test our third hypothesis, we found the rise in domestic abuse to be consistent across the entire lockdown period, declining only when lockdown was eased in May. Examining the later months, the results showed that the probability of being a victim of domestic abuse remained higher than pre-lockdown rates, indicating lockdown has had an enduring effect on domestic abuse beyond the immediate lockdown period for all groups. The probability of being a victim of domestic abuse remained slightly higher than the pre-lockdown levels 3 months after lockdown ends.

The findings portrayed in this paper are consistent with previous research that suggests domestic abuse cases often rise after major incidents (Anastario et al., 2009; Chew et al., 2005; Connell, 2002; Dasgupta et al., 2010; Dobson, 1994; Domeisen, 1998; Fothergill, 1998; Klein, 2008). Like this work, our research has shown that the people that are most at risk are the most vulnerable, women, the young and the old and certain ethnic groups.

## Limitations

Unfortunately, we are unable to elaborate on what it is about lockdown that leads to the increase in domestic abuse. The fact that domestic abuse starts to increase a week before lockdown is instigated may indicate that it is the rise in other stressors related to the wider COVID-19 situation that are dominant factors, rather than the confinement itself. The fact that domestic abuse does not increase with the duration of confinement and does not quickly revert to pre-lockdown levels may also speak to this interpretation. On the other hand, the start of the rise the week before lockdown, which ties in with the start of Boris Johnson’s daily press briefings, may reflect an anticipation effect, with people beginning to confine their movements prior to the formal lockdown decree. This leads to a small increase in domestic abuse almost immediately, which continues to increase over the first week of lockdown. The fact that domestic abuse declines as soon as lockdown conditions are eased in May rather than formally lifted in the middle of June may reflect confinement is the major driver behind the results we have presented here.

## Conclusion and Implications

Despite our inability to elaborate on the underlying mechanisms at work here the results we present produce a clear message for policy makers: When instituting policies aimed at alleviating one problem there is a careful need to think through the implications the policy might have elsewhere. In this case, a policy aimed at reducing the spread of the pandemic, its associated health risks, and alleviating pressure on the NHS had unintended negative consequences for domestic abuse. In most cases of pandemics or other major incidents, these policies will be emergency measures that have to be taken but there needs to be more consideration for what this means for already vulnerable groups, and the possibility that any measures taken may lead to an increase in domestic abuse needs to be factored into any plans at the outset. In this case, the U.K. government’s overall pandemic plan which was published on March 3rd, includes no discussion of domestic abuse (Department of Health and Social Care, 2020), and the National Oversight Group on Domestic Abuse, a cross-party advisory group, did not meet prior to, or during, lockdown. Indeed, the U.K. government only commissioned its first strategic action plan for addressing domestic abuse in late May 2020, 2 months into lockdown. Even at this stage, the report suggested that violence against women and girls was not a key part of the response to the pandemic (New York Times, July 2020). Other countries, such as New Zealand, were seen as having a more proactive response to the inclusion of domestic abuse planning in their COVID-19 response (New York Times, July 2020).

Any policy discussion around this area must include maintaining or even expanding provisions for victims of domestic abuse in times of crisis rather than them being reduced due to repurposing as they have been in some instances during the COVID-19 pandemic. This may include increasing staff or provision to hotlines and outreach centers, providing resources for victims even in lockdown situations, as well as increasing communication and awareness of these services. In lockdown situations, there is a case for making sure one-stop centers remain open and that counseling and support groups continue to function. Where they are shut down, they need to be replaced with virtual options that address inequalities in access to technology (Peterman et al., 2020). Perhaps most importantly, the fact the results show that lockdown exacerbated existing inequalities, with women, the young and older populations, and those of certain ethnic groups most at risk, means that policy responses need to target those most in need. Specialist provision may be needed for children, as they are unlikely to have access to the same outreach resources as adults (Yaker and Erskine, 2020). Likewise, it may take specialist intervention to reach other vulnerable groups identified in this paper, such as those over the age of 70 and those of Asian, Arab and Middle Eastern ethnicity.

## Notes

1. Other countries that have already instituted national lockdown include Italy (March 10th), Spain (March 14th), France (March 16th), Belgium (March 17th), and Argentina (March 21st).

2. Although in the United Kingdom and elsewhere evidence suggests that women are much more likely to be the victims (Home Office, 2020), and are subject to more severe physical violence and sexual violence than men (Smith et al., 2018) and more likely to fear for their safety, miss school or work or sustain injury than male victims (Drotning et al., 2023).

3. Universal Credit is a payment to help with living costs for those who have low income, are out of work, or cannot work for other reasons.

4. We do not include literature from hospital admissions for domestic abuse due to the relatively low number of patients presenting with domestic abuse injuries. For example, Hassan et al. (2020) showed an increase in consultations for plastic surgery-related traumatic injuries as a result of DV in the University of Chicago Trauma Center post-lockdown. However, the numbers were very small, increasing from three to five in the before and after period examined. In the United Kingdom, Murphy et al. (2020) examine the referrals for trauma and orthopedic services in Gloucester Hospital Trust. They found no change in the on-call referrals for domestic abuse-related injuries over the lockdown period but, examining a 4 year period from 2017 to 2020, they found only one referral for domestic abuse-related injuries in 2018. While Olding et al. (2021) found a slight increase in domestic abuse-related injuries at Kings College Hospital, London. But with similarly low numbers (with 1 injury in 2018, 0 in 2019 and 2 in 2020).

5. Kofman et al. (2023) suggest a number of potential explanations for this difference including population density and socio-economic differences across the two areas.

6.
https://www.met.police.uk/foi-ai/metropolitan-police/d/june-2022/domestic-abuse-officers-in-the-mps/#:~:text=Definition%3A%20The%20MPS%20%2F%20ACPO%20%2D,regardless%20of%20gender%20and%20sexuality

7. CRIS reports can come from: (a) Automated alarm message to police (covers all alarm calls); (b) reports direct to officer on duty and away from police building; (c) reports by person calling at police building; (d) discovered by police (i.e., witnessed an event while out on duty); (e) online reporting; (f) reports to police by social services; (g) reports to police by school/education authority; (h) reports to police by dr/hospital; (i) any report from Health Clinic sexual assault unit; (j) reports by means other than above (letter/fax, etc.); (l) phone call to police (999/101); (m) report by email; (n) crime transferred in from another force; (o) reports received from third party report sites; (p) reports to police by fire brigades.

8. Chosen to avoid the Christmas period.

9. This is the analytical sample used for all analyses.

10. The 12 areas are: 1 “Central East,” 2 “Central North,” 3 “Central South,” 4 “Central West,” 5 “East Area,” 6 “North,” 7 “North East,” 8 “North West,” 9 “South,” 10 “South East,” 11 “South West,” 12 “West Area.” We aggregate to this level for the area controls as there are too few hate crimes to analyze the data at Borough level.

11. The AME is the average change in the probability of being a victim of domestic abuse in the lockdown period compared to the period before lockdown. Because the probit is non-linear the relationship will differ for each individual depending on their location in the distribution. The AME calculates a value for each individual with their observed levels of co-variation then computes the average across all observations.

12. Murder and arson are not included as the number of these types of domestic abuse crimes was too low to analyze.

13. Includes both domestic abuse and non-domestic abuse victims.

14. Mobility data suggest that mobility in the United Kingdom began to decrease dramatically prior to the official lockdown order on the 23rd of March 2020 (Google, 2020).
